# miR‐19a‐3p and miR‐19b‐3p Promote Microglia Activation Associated With Neuroinflammation

**DOI:** 10.1002/jnr.70137

**Published:** 2026-06-25

**Authors:** Faezeh Sahebdel, Ricardo A. Battaglino, Leslie R. Morse, Julie K. Olson

**Affiliations:** ^1^ Department of Rehabilitative Medicine, School of Medicine University of Minnesota Minneapolis Minnesota USA; ^2^ Department of Diagnostic and Biological Sciences, School of Dentistry University of Minnesota Minneapolis Minnesota USA; ^3^ Department of Veterinary Biomedical Sciences College of Veterinary Medicine, University of Minnesota Minneapolis Minnesota USA

**Keywords:** microglia, microRNA, neuroinflammation

## Abstract

Neuroinflammation, driven by microglia activation and the production of pro‐inflammatory cytokines, has been implicated in several neurological diseases and neuropathic pain. MicroRNAs (miRNAs) have emerged as important regulators of neuroinflammatory processes. Prior studies identified elevated levels of circulating miR‐19a and miR‐19b in individuals living with chronic pain following spinal cord injury (SCI). In this study, we wanted to determine whether miR‐19a and miR‐19b have a direct effect on microglia activation, specifically pro‐inflammatory activation, associated with neuroinflammation. Microglia were activated by inflammatory stimuli in the presence of miR‐19a or miR‐19b mimics, and assessed for the expression of cytokines, chemokines, and effector molecules. The results show that miR‐19a or miR‐19b mimics increased the expression of pro‐inflammatory cytokines, chemokines, and effector molecules in microglia. The results also showed decreased expression of suppressor of cytokine signaling (SOCS) proteins, namely SOCS1 and SOCS3, in activated microglia with miR‐19a and miR‐19b mimics. Additionally, enhanced signaling through the NFκB and Jak pathways was observed with increased NFkB‐p65 and JAK1 phosphorylation in the presence of miR‐19a and miR‐19b mimics. Further results show that miR‐19a and miR‐19b inhibitors reversed these effects on activated microglia. Overall, our results demonstrate that miR‐19a or miR‐19b increased the expression of pro‐inflammatory cytokines, chemokines, and effector molecules in activated microglia. These results indicate that miR‐19a and miR‐19b can enhance microglia activation and associated inflammatory responses, which may have implications for conditions associated with neuroinflammation.

## Significance Statement

miR19a and miR19b, which are increased in patients with pain following SCI, enhance microglia activation toward a pro‐inflammatory response promoting neuroinflammation.

## Graphical abstract

miR19a and miR19b enhance microglia activation.

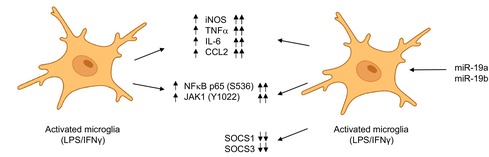

## 1 Introduction

Microglia are known to regulate neuroinflammation, and their activation has been associated with various neurological diseases. Glia cells, including oligodendrocytes and astrocytes, and microglia have also been implicated in neuroinflammation and the development of neuropathic pain. Microglia activation in the spinal dorsal horn has been shown to play a dominant role in neuroinflammation and the pathogenesis of neuropathic pain (Kühlein et al. 2011). Understanding the role of microglia in neuroinflammation and neuropathic pain is crucial for developing new strategies to target these cells and mitigate pain in affected individuals.

MicroRNAs (miRNAs) are a class of small non‐coding RNA molecules, approximately 22 nucleotides in length, that play important roles in regulating gene expression (Griffiths‐Jones 2004). MiRNAs are involved in various cellular processes, including neurogenesis, neuronal survival, dendritic expansion, and spine development, highlighting their significance in the functioning of the nervous system (Kosik 2006). MiRNAs can also influence inflammatory processes and neuroinflammation by modulating the expression of pro‐inflammatory cytokines. Studies have demonstrated that specific miRNAs, such as miR‐155, can increase the expression of pro‐inflammatory cytokines in microglia, contributing to the development of neuroinflammation (Cardoso et al. 2012). Additionally, miRNAs have been implicated in experimental inflammatory pain, with certain miRNAs, such as miR‐133 and let‐7, associated with the development of tolerance to morphine antinociception (He et al. 2010; Sanchez Freire et al. 2010).

Neuroinflammation is characterized by an inflammatory response within the nervous system. Spinal cord injury (SCI) is a debilitating neurological condition that can result in the loss of sensory and motor function below the site of injury (Wilson et al. 2012). Neuroinflammation is commonly observed after SCI and is also associated with chronic pain syndromes that develop in a significant proportion of patients following SCI (Hulsebosch et al. 2009). The relationship between inflammation and pain is intertwined (Walters 2014), and the neuroinflammatory processes that occur after SCI are thought to contribute to the chronic and severe pain experienced by many individuals living with this condition. Understanding the mechanisms underlying neuroinflammation and its association with pain is crucial for the development of effective therapeutic strategies to alleviate pain and improve the quality of life for individuals.

Recent research (Ye et al. 2021) identified 71 differentially expressed miRNAs in individuals with chronic SCI and neuropathic pain compared to those without pain. Two miRNAs, hsa‐miR‐19a‐3p and hsa‐miR‐19b‐3p, were significantly elevated in individuals with SCI‐related neuropathic pain at least one year post injury and exhibited good discriminatory ability between pain and non‐pain groups (Ye et al. 2021). Since microglia activation and neuroinflammation have been associated with chronic pain, the aim of these studies is to determine the effect of miR‐19a and miR‐19b on microglia activation. We wanted to determine whether miR‐19a and miR‐19b can increase the expression of pro‐inflammatory cytokines, chemokines, and effector molecules by microglia promoting neuroinflammation. These studies were conducted with microglia activated through innate immune receptors or activated by inflammatory cytokines. By examining the effects of these miRNAs on microglia, this study may elucidate the effect of miR‐19a and miR‐19b on the pro‐inflammatory response by activated microglia.

## 2 Materials and Methods

### 2.1 Microglia Cultures

Primary microglia cells from SJL/J mice were derived as previously described (Olson et al. 2003, 2001). Brains were removed from male and female neonatal mice and cultured in a mixed glia culture for 14 days. The primary microglia were removed from the flask and resuspended in DMEM (Invitrogen Life Technologies) supplemented with 20% FCS and 3 ng/mL rGM‐CSF (R&D Systems). The cells were then placed on a shaker for 24 h to detach the microglia. Microglia were cultured in poly‐D‐lysine coated 24 well plates with DMEM high‐glucose medium (Gibco), supplemented with 20% FBS and 3 ng/mL rGM‐CSF (R&D Systems). Microglia purity in the culture was greater than 98% as determined by immunostaining the cells with antibody for Iba (microglia) and GFAP (astrocytes).

### 2.2 Microglia Activation and Transfection

Microglia cultures were stimulated with lipopolysaccharides (LPS) (5 μg/mL) (Sigma‐Aldrich) or interferon‐gamma (rIFN‐γ) (2 μg/mL) (Sigma). Microglia were cultured with miRCURY LNA miRNA mimics or miRCURY LNA miRNA inhibitors (Qiagen). Specifically, microglia were transfected with (1) miR‐19a mimic, (2) miR‐19a inhibitor, (3) miR‐19b mimic, (4) miR‐19b inhibitor, and (5) miR mimic control (1 nM) (Qiagen), and then unstimulated, LPS‐stimulated, or IFNγ‐stimulated for 24 h. miRNAs were transfected using Lipofectamine RNAiMAX (Invitrogen) according to the manufacturer's protocol (Qiagen) 4 h prior to stimulation.

### 2.3 RNA Isolation and Real‐Time PCR Analysis

Following the incubation for 24 h, the SV Total RNA Isolation kit (Promega) was used to lyse and isolate total RNA from the microglia cells. The Advantage for RT‐PCR kit (Clontech Laboratories, Takara Bio Company) was used for making cDNA using 2 μg of microglia total RNA by oligo(dT)_12–18_ primers, and the final volume was 100 ul. Rotor‐Gene SYBR Green PCR kit (Qiagen) was used for real‐time PCR. QiagenQ was used for real‐time PCR. First, 40 cycles were done: 15 s in 95°C, 2 s in 60°C, and 15 s in 72°C, then a cycle from 75°C to 95°C, and 5 min in 72°C for the final extension. Primers were used for Il‐6, TNFα, iNOS, CCL2, SOCS1, and SOCS3 (Olson and Miller 2004; Boontanrart et al. 2016). Cells that are known to express the specific cytokines were used as positive control to make cDNAs. β‐Actin was used as the housekeeping gene for normalization between samples.

### 2.4 ELISA and Antibody Array

Following incubation for 24 h, the amount of phosphorylated NFκB p65 Ser536 protein in the microglia cell lysate was measured using commercial mouse‐specific ELISA kits (RayBiotech) according to the manufacturer's instructions. Following incubation for 24 h, the amount of phosphorylated JAK1 Y1022 protein in the microglia cell lysate was measured using antibody array assay technique (Full Moon Biotech).

### 2.5 Statistical Analysis

Three biological duplicates were carried out for all the experiments. Real‐time PCR was performed in triplicate (technical replicate) and results were calculated by the mean ± SD. Significant differences between stimulated and unstimulated microglia groups were conducted using a one‐way ANOVA analysis and Bonferroni comparison test. For the analyses, a *p*‐value lower than 0.05 was considered statistically significant.

## 3 Results

### 3.1 miR‐19a and miR‐19b Increased the Pro‐Inflammatory Response in Microglia

**FIGURE 1:**
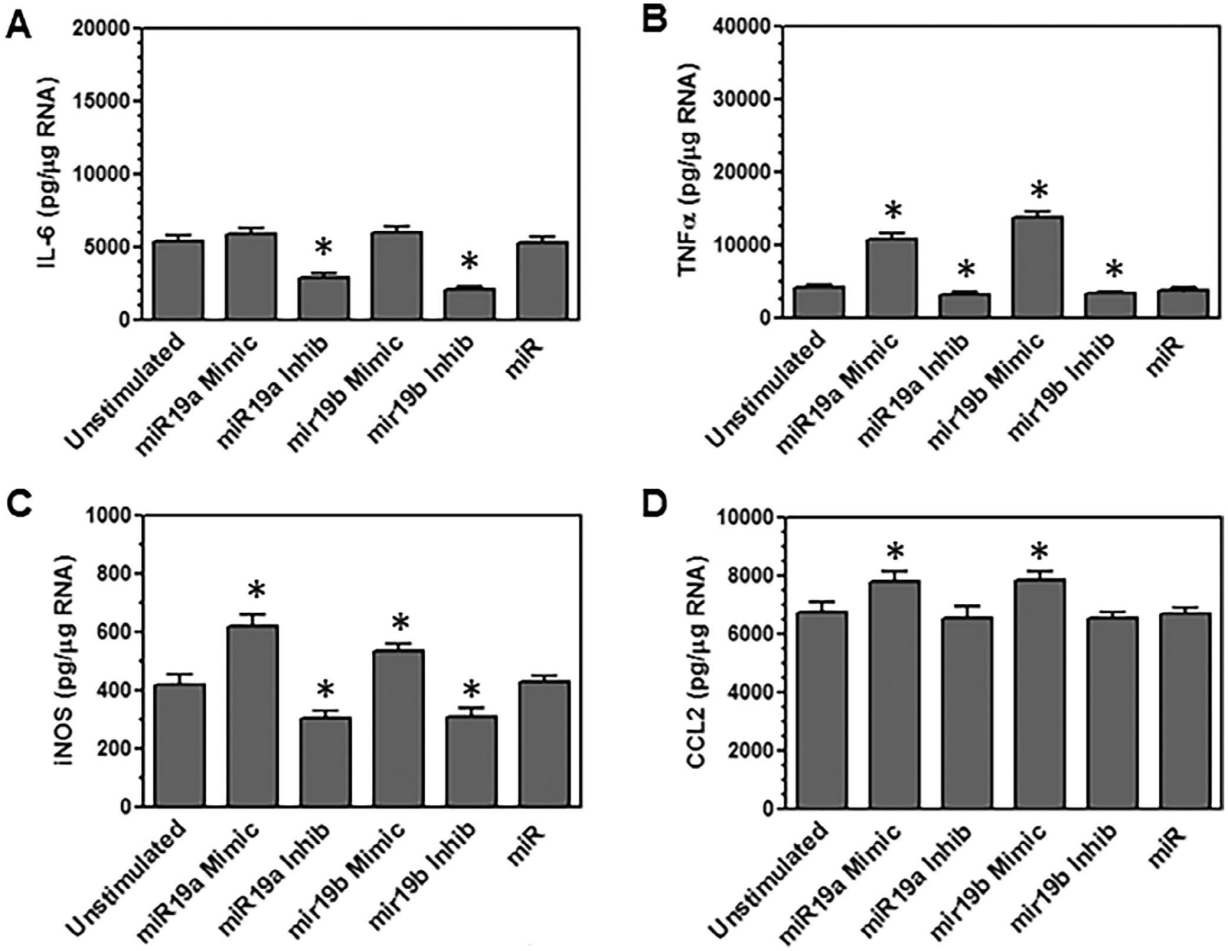
miR‐19a and miR‐19b increased the expression of pro‐inflammatory cytokines, chemokines, and effector molecules in microglia. Microglia were transfected with miR‐19a mimic, miR‐19b mimic, or negative control miR mimic, or microglia were transfected with miR‐19a inhibitor or miR‐19b inhibitor. After 24 h, microglia were lysed and RNA was isolated. RNA was converted to cDNA and used in real‐time PCR with primers for[A] IL‐6, [B] TNF‐α, [C] iNOS, and [D]CCL2. The concentration was based on standards for each set of primers, and groups were normalized based on the expression of β‐actin. Significant difference (*) was determined by one way anova and Bonferroni's multiple comparison test (*p < 0.001) based on unstimulated microglia. These are representative graphs from one experiment of three independent repeated experiments.

Our previous studies showed that miR‐19a and miR‐19b were increased in SCI patients with pain (Ye et al. 2021). Previous studies have shown that chronic pain is associated with neuroinflammation mediated by microglia in the CNS. Thus, we wanted to determine whether miR‐19a and miR‐19b have a direct effect on microglia activation, specifically pro‐inflammatory activation, associated with neuroinflammation. Microglia were transfected with miR‐19a mimic, miR‐19b mimic, negative control miR mimic, miR‐19a inhibitor, or miR‐19b inhibitor. Microglia were then examined for the expression of cytokines, chemokines, and effector molecules. miR‐19a mimic increased the expression of TNFα, iNOS, and CCL2 in microglia compared to control treated microglia (Figure 1). On the other hand, miR‐19a inhibitor decreased the expression of IL‐6, TNFα, and iNOS in microglia compared to control treated microglia (Figure 1). miR‐19b mimic increased the expression of TNFα, iNOS, and CCL2 in microglia while miR‐19b inhibitor decreased the expression of IL‐6, TNFα, and iNOS compared to control treated microglia. These results show that miR‐19a mimic and miR‐19b mimic both similarly increased the expression of pro‐inflammatory cytokines, chemokines, and effector molecules in microglia while miR‐19a inhibitor and miR‐19b inhibitor decreased the expression of pro‐inflammatory cytokines, chemokines, and effector molecules.

### 3.2 miR‐19a and miR‐19b Increased the Pro‐Inflammatory Response in LPS‐Stimulated Microglia

**FIGURE 2:**
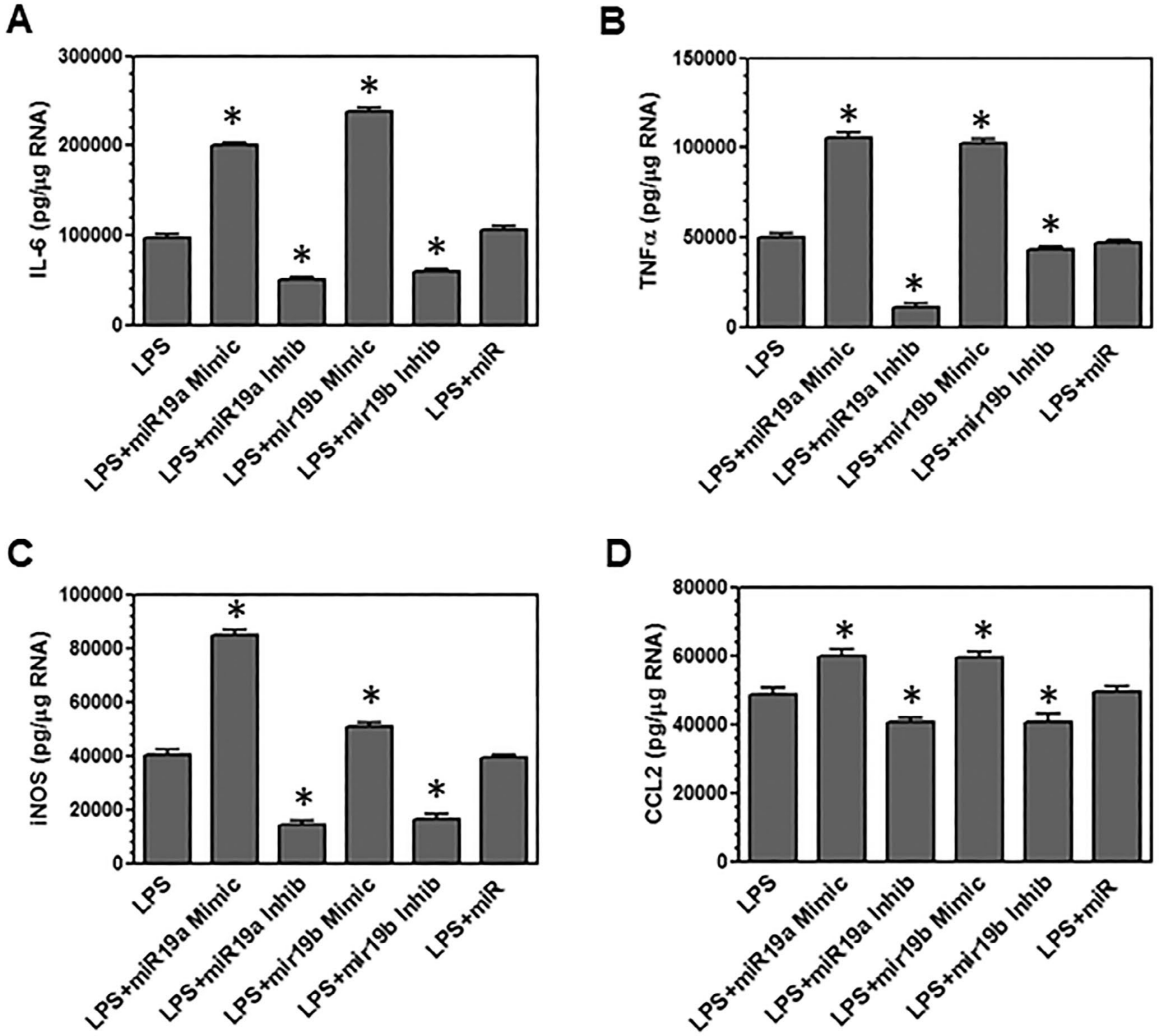
miR‐19a and miR‐19b increased the expression of pro‐inflammatory cytokines, chemokines, and effector molecules in LPS‐stimulated microglia. Microglia were stimulated with LPS (5 μg/mL) and transfected with miR‐19a mimic, miR‐19b mimic, negative control miR mimic, miR‐19a inhibitor, or miR‐19b inhibitor. After 24 h, microglia were lysed and RNA was isolated. RNA was converted to cDNA and used in real‐time PCR with primers for [A] IL‐6, [B] TNF‐α, [C] iNOS, or [D] CCL2. The concentration was based on standards for each set of primers, and groups were normalized based on the expression of β‐actin. Significant difference (*) was determined by one way anova and Bonferroni's multiple comparison test (*p < 0.001) based on LPS‐stimulated microglia. These are representative graphs from one experiment of three independent repeated experiments.

Following SCI, microglia can be activated by damage associated molecular patterns (DAMPs) which are recognized by Toll like receptors (TLRs) (Olson and Miller 2004). High‐mobility group box 1 (HMGB1) is a nonhistone chromatin binding protein that can be released from damaged cells as a DAMPs and has been shown to be released following SCI (Chen et al. 2011). HMGB1 is recognized by TLR4 resulting in an inflammatory response (Zhang et al. 2015). Thus, we wanted to determine the effect of miR‐19a and miR‐19b on microglia activated through TLRs. LPS activates microglia through TLR4 which is similar to HMGB1 (Zhang et al. 2015). Microglia were stimulated with LPS and transfected with miR‐19a mimic, miR‐19b mimic, or negative control miR mimic. Additional microglia were stimulated with LPS and transfected with miR‐19a inhibitor or miR‐19b inhibitor. After 24 h later, microglia were analyzed for the expression of cytokines, chemokines, or effector molecules. LPS‐stimulated microglia increased the expression of the IL‐6, TNFα, iNOS, and CCL2 (Figure 2). miR‐19a mimic and miR‐19b mimic significantly increased the expression of IL‐6, TNFα, iNOS, and CCL2 by the LPS‐stimulated microglia. On the contrary, miR19a inhibitor and miR19b inhibitor decreased the expression of IL‐6, TNFα, iNOS, and CCL2 by the LPS‐stimulated microglia (Figure 2). The control miR mimic transfected LPS‐stimulated microglia had similar expression of the cytokines, chemokines, and effector molecules as the LPS‐stimulated microglia that were not transfected (Figure 2). The expression of miR‐19a and miR‐19b was not significantly increased with LPS‐stimulation alone as compared to unstimulated microglia (Figure S1). These results show that miR‐19a and miR‐19b increased the expression of pro‐inflammatory cytokines, chemokines, and effector molecules in microglia activated through TLRs.

### 3.3 miR‐19a and miR‐19b Mimics Increased the Pro‐Inflammatory Response in IFNγ‐Stimulated Microglia

**FIGURE 3:**
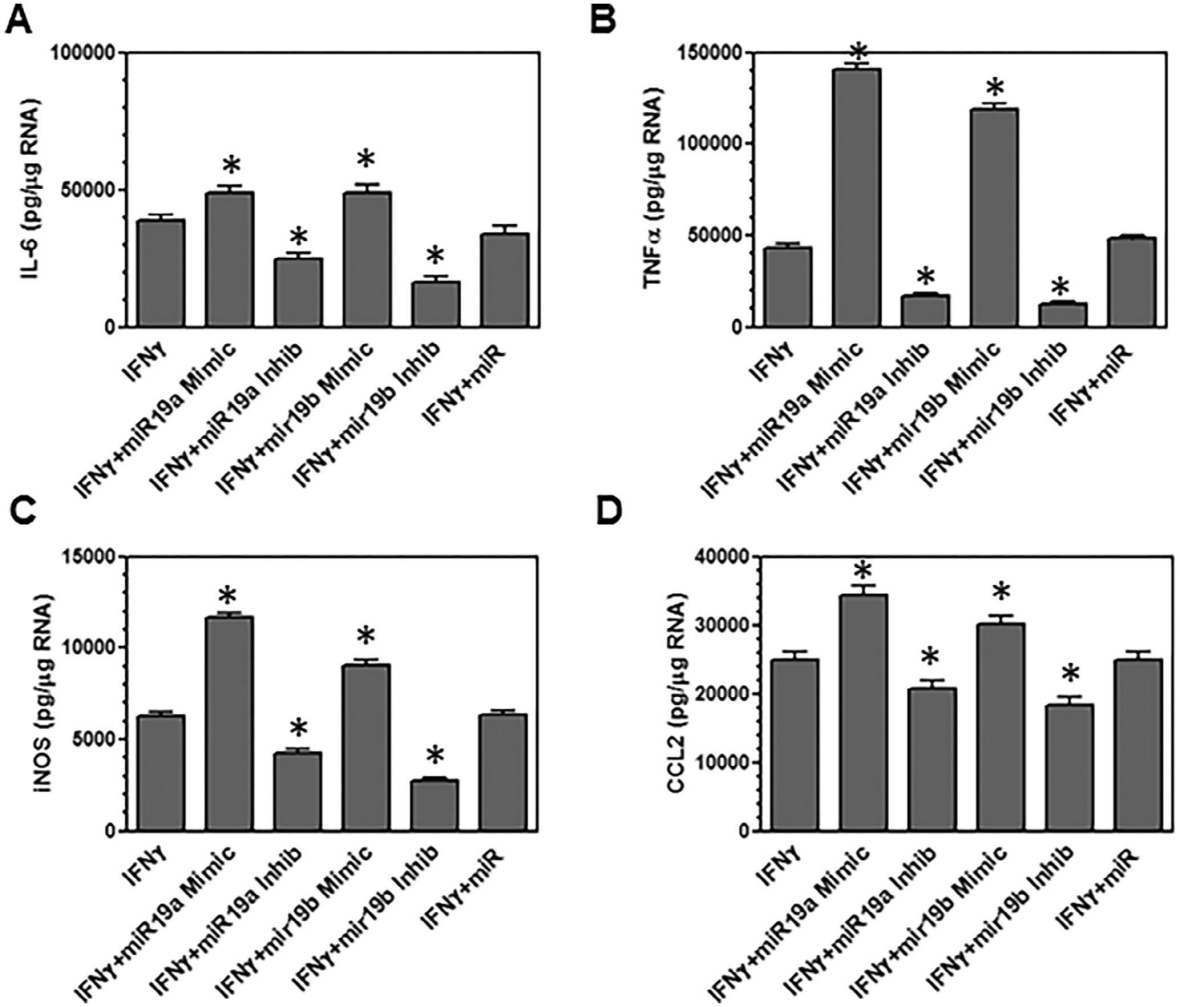
miR‐19a and miR‐19b increased the expression of pro‐inflammatory cytokines, chemokines, and effector molecules in IFNγ‐stimulated microglia. Microglia were stimulated with IFNγ (100 U/mL) and transfected with miR‐19a mimic, miR‐19b mimic, negative control miR mimic, miR‐19a inhibitor, or miR‐19b inhibitor. After 24 h, microglia were lysed and RNA was isolated. RNA was converted to cDNA and used in real‐time PCR with primers for [A] IL‐6, [B]TNF‐α, [C] iNOS, or [D] CCL2. The concentration was based on standards for each set of primers, and groups were normalized based on the expression of β‐actin. Significant difference (*) was determined by one way anova and Bonferroni's multiple comparison test (*p < 0.001) based on IFNγ‐stimulated microglia. These are representative graphs from one experiment of three independent repeated experiments.

Following SCI and during neurological diseases, immune cells from the periphery infiltrate to the site of the injury. These infiltrating immune cells including T cells can secrete IFNγ which has been shown to activate microglia (Olson et al. 2001). We wanted to determine how miR‐19a and miR‐19b would affect IFNγ‐stimulated microglia. IFNγ‐stimulated microglia were transfected with miR‐19a mimic, miR‐19b mimic, negative control miR mimic, miR‐19a inhibitor, or miR‐19b inhibitor. After 24 h, microglia were analyzed for the expression of cytokines, chemokines, and effector molecules. IFNγ‐stimulated microglia had increased expression of IL‐6, TNFα, iNOS, and CCL2 compared to control treated microglia (Figure 3). miR‐19a mimic and miR‐19b mimic increased the expression of IL‐6, TNFα, iNOS, and CCL2 in the IFNγ‐stimulated microglia. The expression of miR‐19a and miR‐19b was not significantly increased with IFNγ‐stimulation alone as compared to unstimulated microglia (Figure S1). Meanwhile, miR‐19a inhibitor and miR‐19b inhibitor decreased the expression of IL‐6, TNFα, iNOS, and CCL2 in IFNγ‐stimulated microglia (Figure 3). These results show that miR‐19a and miR‐19b increased the expression of pro‐inflammatory cytokines, chemokines, and effector molecules in microglia activated by IFNγ.

### 3.4 miR‐19a and miR‐19b Reduced Expression of SOCS1 and SOCS3 in Microglia

**FIGURE 4:**
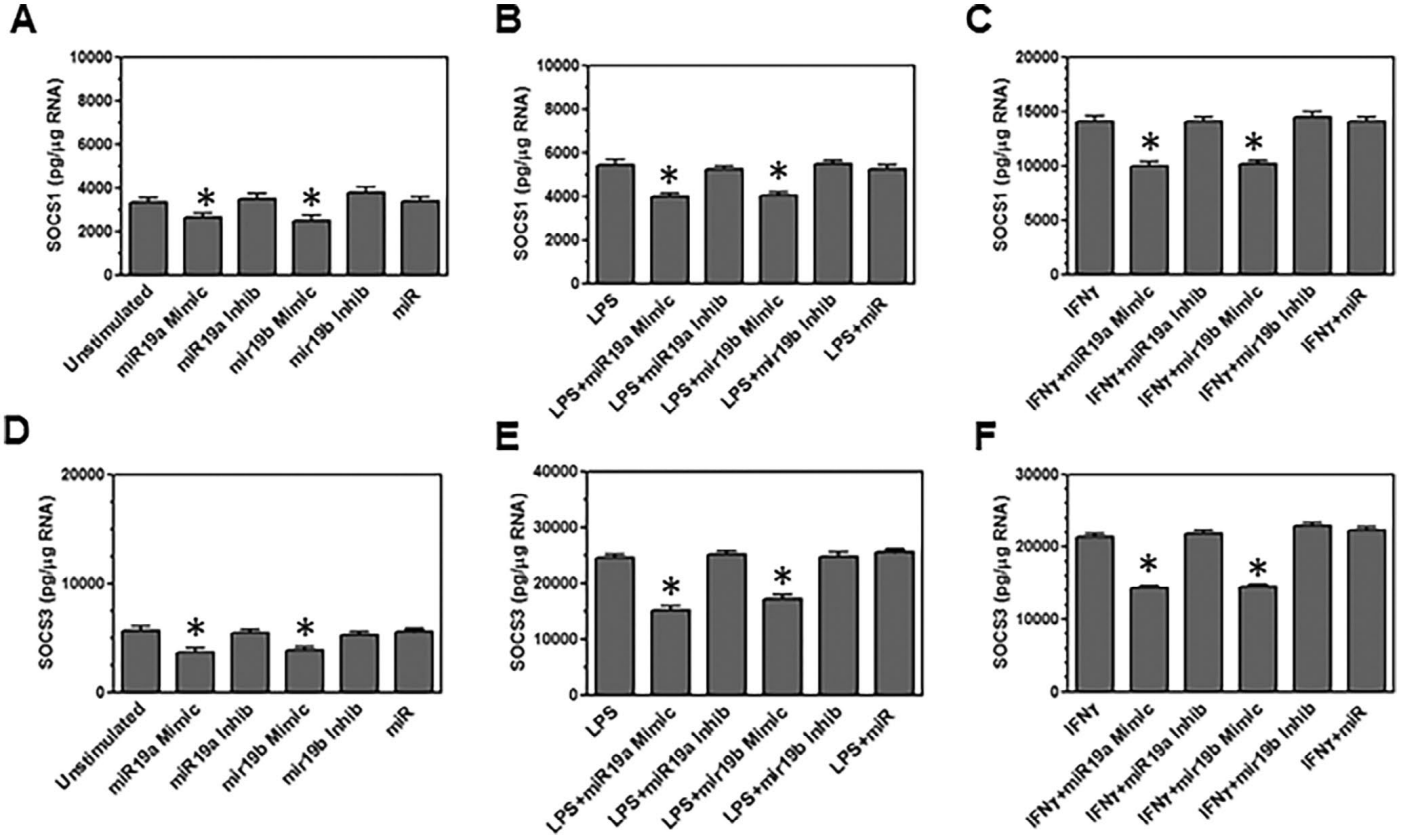
miR‐19a and miR‐19b decreased the expression of SOCS1 and SOCS3 in microglia. Microglia were unstimulated (A, D), or stimulated with LPS (B, E), or stimulated with IFNγ (C, F). Microglia were also transfected with miR‐19a mimic, miR‐19b mimic, negative control miR mimic, miR‐19a inhibitor, or miR‐19b inhibitor. After 24 h, microglia were lysed and RNA was isolated. RNA was converted to cDNA and used in real‐time PCR with primers for SOCS1 (A–C) and SOCS3 (D–F). The concentration was based on standards for each set of primers, and groups were normalized based on the expression of β‐actin. Significant difference (*) was determined by one way anova and Bonferroni's multiple comparison test (*p < 0.001) based on unstimulated microglia, LPS‐stimulated microglia, or IFNγ‐stimulated microglia. These are representative graphs from one experiment of three independent repeated experiments.

Suppressor of cytokine signaling 1 (SOCS1) and SOCS3 are negative regulators of pro‐inflammatory cytokine expression. miR19a and miR19b have been shown to bind to the 3′ untranslated region of SOCS1 and SOCS3 (Wen et al. 2025; Collins et al. 2013; Shi et al. 2022; Chen et al. 2015). We wanted to determine whether miR‐19a and miR‐19b can decrease the expression of SOCS1 and SOCS3 in activated microglia correlating with increased expression of pro‐inflammatory cytokines. Microglia were stimulated with LPS or IFNγ and transfected with miR‐19a and miR‐19b mimics or inhibitors. SOCS1 and SOC3 expression was determined after 24 h. SOCS 1 expression was decreased in naïve microglia transfected miR‐19a mimic or miR‐19b mimic (Figure 4). SOCS1 expression is increased when microglia are activated, including LPS and IFNγ‐stimulation. However, miR‐19a mimic and miR‐19b mimic decreased the expression of SOCS1 in LPS‐stimulated microglia and IFNγ‐stimulated microglia (Figure 4). miR‐19a inhibitor and miR‐19b inhibitor did not significantly affect the expression of SOCS1. SOCS3 expression was significantly reduced in naïve microglia transfected with miR‐19a mimic and miR‐19b mimic (Figure 4). SOCS3 expression is increased when microglia are activated, LPS and IFNγ stimulation. Similarly, miR‐19a mimic and miR‐19b mimic decreased the expression of SOCS3 in LPS‐stimulated and IFNγ‐stimulated microglia. SOC3 expression was not significantly different in microglia transfected with miR‐19a inhibitor or miR‐19b inhibitor. These results show the miR‐19a and miR‐19b decrease the expression of SOCS1 and SOCS3 in both unstimulated and stimulated microglia.

### 3.5 miR‐19a and miR‐19b Mimics Increased Phosphorylation of NFκB p65 in LPS‐Stimulated Microglia

**FIGURE 5:**
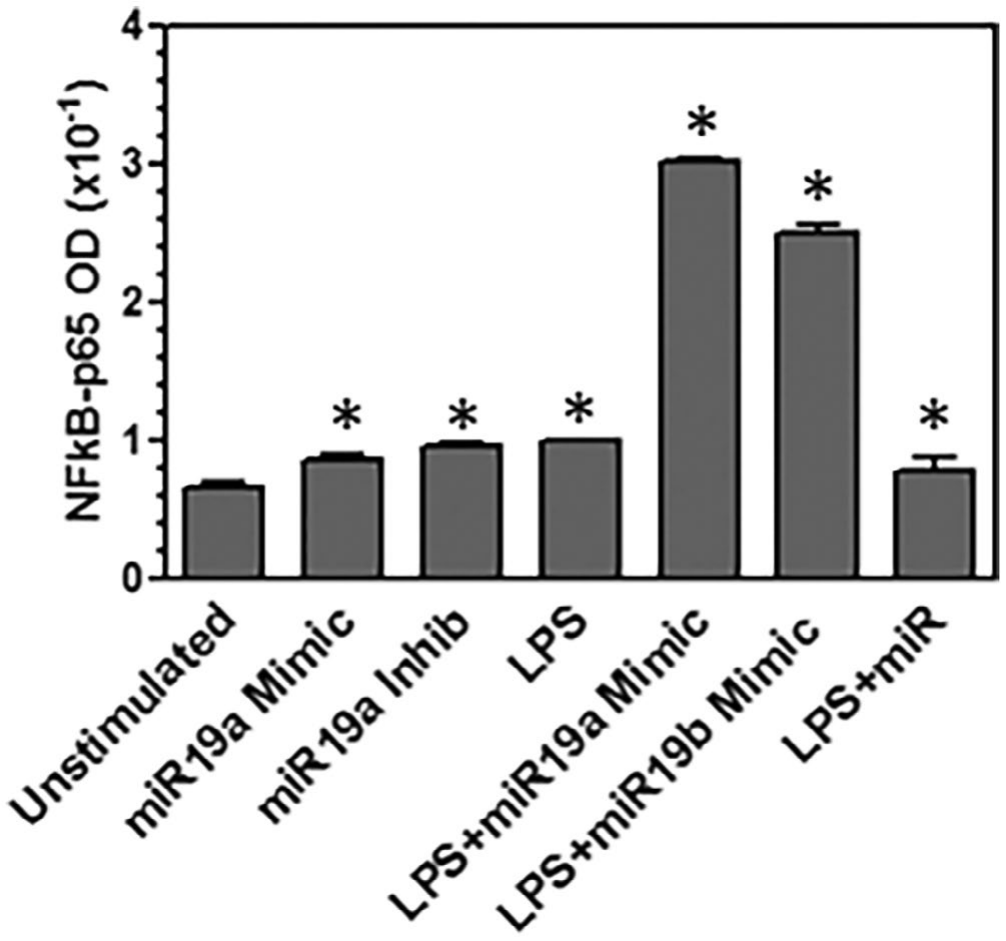
miR‐19a and miR‐19b increased phosphorylation of NFκB p65 in microglia. Microglia were transfected with miR‐19a mimic or miR‐19b mimic. Microglia were stimulated with LPS and transfected with miR‐19a mimic, miR‐19b mimic, or negative control miR mimic. After 24 h, the microglia were lysed and whole protein extracts were analyzed by ELISA for P65 (serine 536). Significant difference (*) was determined by one way anova and Bonferroni's multiple comparison test (*p < 0.001) based on unstimulated microglia. These are representative graphs from one experiment of three independent repeated experiments.

Activation of microglia through TLR4 with LPS leads to the activation of NFκB. We wanted to determine whether miR‐19a and miR‐19b alter the activation of the NFκB pathway leading to increased expression of pro‐inflammatory cytokines, chemokines, and effector molecules in LPS‐stimulated microglia. NFκB p65 is phosphorylated on serine 536 to activate the NFκB complex to promote transcription. Microglia were unstimulated or stimulated with LPS and transfected with miR‐19a mimic or miR‐19b mimic. After 24 h, proteins were analyzed for phosphorylated NFκB p65. miR‐19a and miR‐19b slightly increased the amount of phosphorylated NFκB p65 in unstimulated microglia (Figure 5). LPS‐stimulated microglia had increased levels of phosphorylated NFκB p65, which were greatly increased with miR‐19a and miR‐19b. The results showed that miR‐19a and miR‐19b increased phosphorylation of the NFκB p65 subunit on serine 536 in unstimulated microglia and more significantly in LPS‐stimulated microglia.

### 3.6 miR‐19a and miR‐19b Mimics Increased Phosphorylation of JAK1 in IFNγ‐Stimulated Microglia

**FIGURE 6:**
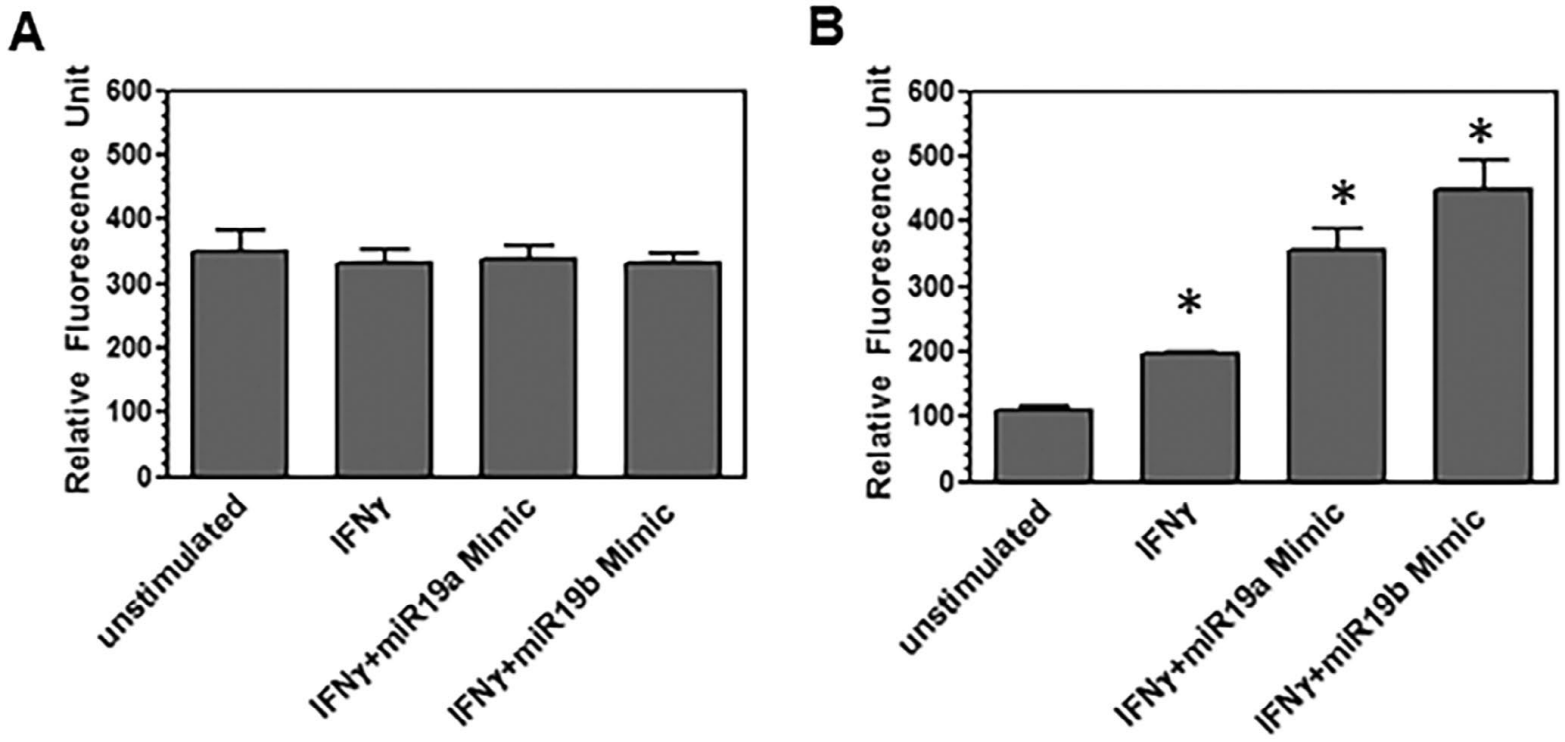
miR‐19a and miR‐19b increased phosphorylation of JAK1. Microglia were unstimulated or stimulated with IFNγ and transfected with miR‐19a mimic or miR‐19b mimic. After 24 h, the microglia were lysed and whole protein extracts were analyzed by Antibody array Assay for total [A] JAK1 and [B] phosphorylated JAK1 (tyrosine 1022). Significant difference (*) was determined by one way anova and Bonferroni's multiple comparison test (*p < 0.001) based on unstimulated microglia. These are representative graphs from one experiment of three independent repeated experiments.

IFNγ stimulation activates the JAK/STAT signaling pathways in cells. JAK1 is activated by phosphorylation on tyrosine 1022 to promote transcription of pro‐inflammatory cytokines, chemokines, and effector molecules. We wanted to determine if miR‐19a and miR‐19b enhance the pro‐inflammatory activation of IFNγ‐stimulated microglia through promoting the phosphorylation of JAK1. Microglia were unstimulated or stimulated with IFNγ and transfected with miR‐19a mimic or miR‐19b mimic. After 24 h, proteins were analyzed for JAK1 and phosphorylated JAK1. Microglia and IFNγ‐stimulated microglia had the same levels of total JAK1 (Figure 6). miR‐19a and miR‐19b did not alter the amount of total JAK1 in IFNγ‐stimulated microglia. On the contrary, IFNγ‐stimulated microglia had an increased amount of phosphorylated JAK1 which was further increased with miR‐19a and miR‐19b (Figure 6). These results show that miR‐19a and miR‐19b promote increased phosphorylation of JAK1 on tyrosine 1022 in IFNγ‐stimulated microglia.

## 4 Discussion

Previous studies showed that miR‐19a and miR‐19b were increased in SCI patients with chronic pain (Ye et al. 2021). Chronic pain has been associated with neuroinflammation mediated by activated microglia. In this study, we investigated the effect of miR‐19a and miR‐19b on microglial activation. These studies show that miR‐19a and miR‐19b mimics increased the expression of pro‐inflammatory cytokines, chemokines, and effector molecules in unstimulated microglia. Following injury and during neurological diseases, microglia can become activated. Infiltrating immune cells, including T cells, can secrete IFNγ, which has been shown to activate microglia (Olson et al. 2001). Microglia can also be activated by PAMPs and DAMPs, which are recognized by innate immune receptors, including Toll‐like receptors (TLRs) (Olson and Miller 2004). LPS was used in these studies to activate microglia through TLR4. Damage‐associated molecule, HMGB1, which can be released following injury, has been shown to activate through TLR4 similar to LPS. These studies show that both LPS and IFN‐γ activated microglia showed increased expression of pro‐inflammatory cytokines, IL‐6 and TNFα, chemokines, CCL2, and effector molecules, iNOS, in the presence of miR‐19a and miR‐19b mimics. Meanwhile, miR‐19a inhibitor and miR‐19b inhibitor decreased the expression of pro‐inflammatory cytokines, chemokines, and effector molecules in activated microglia. These results show that miR‐19a and miR‐19b enhance microglial activation toward a pro‐inflammatory response associated with neuroinflammation.

Nuclear factor‐κB (NFκB) is a transcription factor that plays important roles in inflammation, immunity, cell proliferation, differentiation, and survival. NFκB is activated by LPS binding to TLR4. The Janus Kinase/Signal Transducers and Activators of Transcription (JAK/STAT) signaling pathway is triggered by a variety of cytokines and interferons. The JAK/STAT pathway is required for the development and function of both innate and adaptive immune responses. Given that more than seventy distinct cytokines and interferons employ this channel, it is currently acknowledged that this is one of the most critical pathways for the growth and function of cells that mediate innate and adaptive immunity (Yan et al. 2018). Previous studies have shown that IFNγ stimulation activates the JAK/STAT signaling pathways. JAK1 is activated by phosphorylation on tyrosine 1022 to promote transcription of pro‐inflammatory cytokines, chemokines, and effector molecules. Our studies show that miR‐19a mimic and miR‐19b mimic induced greater phosphorylation of the NFκB p65 subunit on serine 536 and induced phosphorylation of JAK1 on tyrosine 1022 that can increase the expression of pro‐inflammatory cytokines, chemokines, and effector molecules.

Neuroinflammation is regulated by members of the suppressor of cytokine signaling (SOCS) family such as SOCS1 and SOCS3 in microglia cells (Baker et al. 2009; Qin et al. 2006, 2007; Park et al. 2014). Expression of SOCS1 and SOCS3 in microglia controls neuroinflammation by decreasing cytokine expression and nitric oxide production (Park et al. 2003). Several mechanisms regulate SOCS family expression in microglia, including microRNAs (miRNAs). In microglia, microRNAs can regulate neuroinflammation by modulating the expression of SOCS family members (Ye et al. 2021). Some miRNAs can inhibit the expression of suppressor of cytokines leading to an increase in the pro‐inflammatory cytokines and neuroinflammation. For instance, miR‐155 downregulates the expression of SOCS1 and causes an increase in the pro‐inflammatory cytokines in microglia cells (Cardoso et al. 2012). miR‐19a and miR‐19b have been shown to bind to the 3′ untranslated region of SOCS1 and SOCS3 (Wen et al. 2025; Collins et al. 2013; Shi et al. 2022; Chen et al. 2015). NFκB stimulates the expression of many pro‐inflammatory cytokines and chemokines. Members of the SOCS family modulate neuroinflammation by exerting an anti‐inflammatory effect, in part, by suppressing NFκB signaling (Strebovsky et al. 2011). SOCS1 interacts with the p65 subunit, inhibiting the induction of NFκB‐dependent genes. The results from the current studies show that SOCS1 and SOCS3 expression was decreased by miR‐19a and miR‐19b mimics in unstimulated and stimulated microglia. The results also show that the addition of miR‐19a and miR‐19b mimics resulted in increased phosphorylation of p65 for NFκB activation in unstimulated and LPS‐stimulated microglia. However, miR‐19a and miR‐19b have several possible targets that could also be modulating the inflammatory response (Sahebdel et al. 2024).

Cytokine‐mediated cell activation occurs through the JAK/STAT pathway. The kinase activity of cytoplasmic, receptor associated JAKs is initiated when a cytokine attaches to its receptor on the cell membrane (Benveniste et al. 2014). SOCS proteins operate by binding to phosphorylated tyrosine residues on Janus kinases (JAKs) and/or cytokine receptor subunits, interrupting the traditional JAK/STAT signaling cascade by increasing the proteasomal degradation of active receptors and eliminating the stimulus for ongoing activation. Microglia can initiate inflammatory response through the JAK–STAT/SOCS signaling system (Porro et al. 2019). Therefore, the JAK–STAT signaling pathway is an important signaling pathway in neuroinflammatory responses in microglia. The results from the current study show that SOCS1 and SOCS3 expression were decreased by miR‐19a and miR‐19b mimics in unstimulated and stimulated microglia. The results further show that miR‐19a and miR‐19b mimics led to increased phosphorylation of JAK1 on tyrosine 1022 in IFNγ‐stimulated microglia. These results correlate with the increased expression of pro‐inflammatory cytokines, chemokines, and effector molecules in activated microglia.

Previous studies showed that miRNAs have a modulatory effect on neuroinflammation. miRNA‐146a is an inflammatory negative regulator expressed in neurons, microglia, and astrocytes that is also activated by the NF‐κB signaling pathway (Li et al. 2011; Taganov et al. 2006). miRNA‐155 inhibits SOCS1 and SOCS3 signaling, which allows intracellular cytokine signaling to be amplified (Ji and Gattinoni 2015). miRNA‐155 enhances JAK–STAT pathway activation by specifically targeting the repressors SOCS1 and SHIP1. SCI results in extended activation of microglia in the spinal cord, which contributes to the maintenance of neuronal hyperactivity and pain‐related behaviors (Colwell et al. 2006). Inflammation and pain are closely related, and the neuroinflammatory effects of SCI contribute to the chronic pain that can occur in patients following SCI (Walters 2014). Our previous study identified miR‐19a and miR‐19b as upregulated in SCI patients with chronic pain. The current studies show that miR‐19a and miR‐19b enhance the pro‐inflammatory response in microglia which are key mediators of neuroinflammation associated with chronic pain.

## Declaration of Transparency

The authors, reviewers and editors affirm that in accordance to the policies set by the *Journal of Neuroscience Research*, this manuscript presents an accurate and transparent account of the study being reported and that all critical details describing the methods and results are present.

## Author Contributions

J.K.O. and R.A.B. conceived the idea. J.K.O. developed the experimental design. J.K.O. and F.S. conducted the experiments. J.K.O. analyzed the data and organized results. J.K.O. and F.S. wrote the manuscript. R.A.B. and L.R.M. reviewed the manuscript.

## Funding

This work was supported by Departement of Defense (W81XWH‐10‐1‐1043), National Institutes of Health (R01AR064793 and 90SI5015‐01‐00), Paralyzed Veterans of America (3193).

## Conflicts of Interest

The authors declare no conflicts of interest.

## Supporting information


**Figure S1:** miR‐19a and miR‐19b expression was not significantly changed with LPS or IFNγ‐stimulation. Microglia were unstimulated or stimulated with IFNγ or LPS. After 24 h, microglia were lysed and RNA was isolated. RNA was converted to cDNA and used in real‐time PCR with primers for miR‐19a (A) and miR‐19b (B). The concentration was based on standards for each set of primers, and groups were normalized based on the expression of β‐actin. Significant difference (*) was determined by one way anova and Bonferroni's multiple comparison test (*p < 0.001) based on unstimulated microglia.

**Data S1:** Transparent Science Questionnaire for Authors

## Data Availability

The data that support the findings of this study are available from the corresponding author upon reasonable request.
