# Supplementary figures and images for: miR‐19a‐3p and miR‐19b‐3p Promote Microglia Activation Associated With Neuroinflammation
Source: J Neurosci Res. 2026 Jun 25;104(7):e70137. doi: 10.1002/jnr.70137 (PMC13295151; doi:10.1002/jnr.70137)

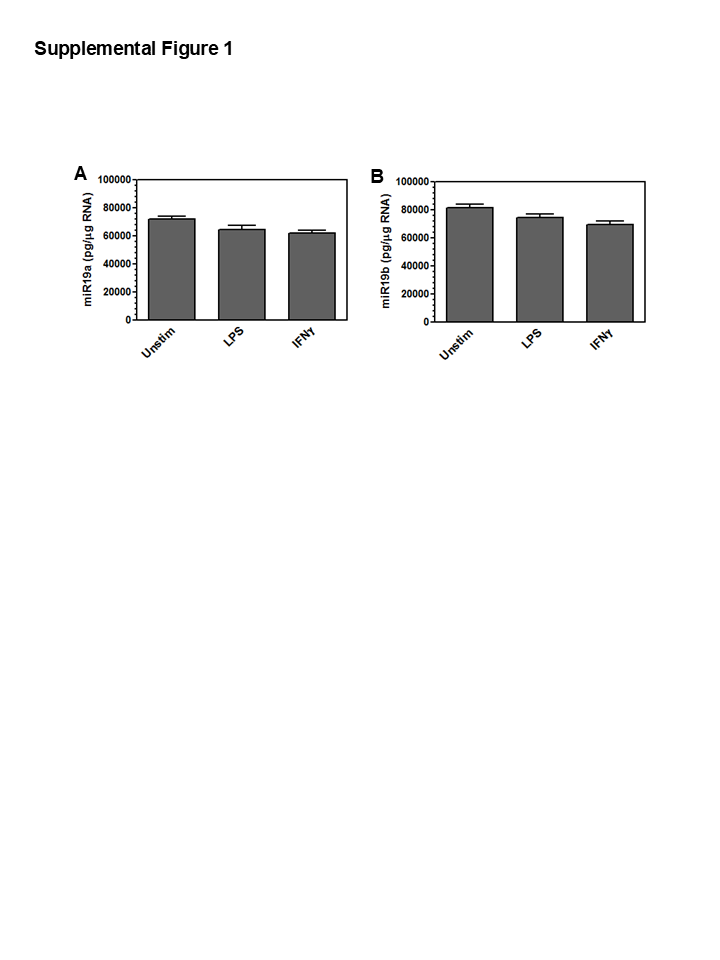

Supplement: Supplementary file 1 — Figure S1: miR‐19a and miR‐19b expression was not significantly changed with LPS or IFNγ‐stimulation. Microglia were unstimulated or stimulated with IFNγ or LPS. After 24 h, microglia were lysed and RNA was isolated. RNA was converted to cDNA and used in real‐time PCR with primers for miR‐19a (A) and miR‐19b (B). The concentration was based on standards for each set of primers, and groups were normalized based on the expression of β‐actin. Significant difference (*) was determined by one way anova and Bonferroni's multiple comparison test (*p < 0.001) based on unstimulated microglia. [file JNR-104-e70137-s001.tif]
